# Oviductal Extracellular Vesicles Enhance Porcine In Vitro Embryo Development by Modulating the Embryonic Transcriptome

**DOI:** 10.3390/biom12091300

**Published:** 2022-09-15

**Authors:** Agostinho Soares de Alcântara-Neto, Cristina Cuello, Rustem Uzbekov, Stefan Bauersachs, Pascal Mermillod, Carmen Almiñana

**Affiliations:** 1 Physiology of Reproduction and Behaviors (PRC), UMR085, INRAE, CNRS, University of Tours, 37380 Nouzilly, France; 2 Department of Animal Medicine and Surgery, Faculty of Veterinary Medicine, Regional Campus of International Excellence, Campus de Espirando, University of Murcia, 30100 Murcia, Spain; 3 Laboratoire Biologie Cellulaire et Microscopie Electronique, Faculté de Médecine, University of Tours, 10 Boulevard Tonnellé, 37200 Tours, France; 4 Faculty of Bioengineering and Bioinformatics, Moscow State University, 119992 Moscow, Russia; 5 Functional Genomics Group, Institute of Veterinary Anatomy, Vetsuisse Faculty, University of Zurich, 8057 Lindau, ZH, Switzerland

**Keywords:** oviductal extracellular vesicles, extracellular vesicles, exosomes, oviductal fluid, embryo development, in vitro embryo production, in vitro culture and porcine

## Abstract

Oviductal extracellular vesicles (oEVs) have been identified as important components of the oviductal fluid (OF) and have been pointed to as key modulators of gamete/embryo-maternal interactions. Here, we determined the functional impact of oEVs on embryo development and the embryonic transcriptome in porcine. Experiment 1 examined the effect of oEVs and OF on embryo development. In vitro-produced embryos were cultured with oEVs or OF for 2 or 7 days using an in vitro sequential system or without supplementation (control). Experiment 2 analyzed transcriptomic alterations of EV-treated embryos versus control and the oEVs RNA cargo by RNA-sequencing. Two days of EV treatment enhanced embryo development over time when compared to other treatments. Different RNA expression profiles between embryos treated with EVs for two or seven days and untreated controls were obtained, with 54 and 59 differentially expressed (DE) genes and six and seven DE miRNAs, respectively. In oEV RNA cargo, 12,998 RNAs and 163 miRNAs were identified. Integrative analyses pointed to specific oEV components that might act as modulators of the embryonic transcriptome, such as *S100A11*, *ANXA2* or miR-21-5p. Overall, the findings suggested that oEVs could be a potential strategy to improve porcine IVP outcomes, particularly by using two days of EV treatment.

## 1. Introduction

The use of reproductive biotechnologies bypasses the early maternal interactions in the oviduct [1]. This lack of maternal–embryo communication affects embryo development and results in embryos of a poor quality [2]. Understanding the beneficial effects of maternal factors that positively regulate and support the embryo might be key to overcome the negative effects of reproductive biotechnologies and poor embryo quality. These maternal factors might differ among species, since each species has its unique features during the early reproductive events (e.g., ovulation of a mature or immature oocyte, time in the oviduct until migration to the uterus). Moreover, unveiling the specific maternal factors contributing to embryo development will bring new clues to enhance the application of the different reproductive biotechnologies.

In porcine, the overall efficiency of in vitro embryo production (IVP) is still extremely low when compared to other species [3]. These limitations have been mainly associated with: (1) an incomplete maturation of the porcine oocyte after in vitro maturation (IVM); (2) a high polyspermy incidence, caused by the simultaneous penetration of one oocyte by several spermatozoa after in vitro fertilization (IVF); (3) a low embryo developmental rate and poor quality of blastocyst at the end of in vitro embryo culture (IVC) due to suboptimal IVC conditions [4,5]. Considering that porcine in vitro produced embryos have become essential materials to study human diseases [6] because of their similarities in body size, physiology, and pathophysiological responses with humans, there is an urge to improve the IVP systems in this species.

To overcome each of the problems related to porcine IVP, extensive research has been performed in the last two decades by improving each single step of the IVP, IVM, IVF or IVC procedures mainly by using different supplements. Since the incidence of polyspermy fertilization in porcine is high when compared to other species, most efforts have been directed to optimize IVF [3,7,8,9,10,11,12,13,14,15]. Only a few studies have investigated the effect of IVC conditions on the embryo development [16,17] and their impact on the embryonic transcriptome [18,19,20]. The IVC medium is meant to mimic the maternal milieu and support the very early embryo development. However, embryos are placed in static IVC media droplets in most IVP media for a week, rather than the in vivo dynamic and interactive maternal milieu. Only a few laboratories have used a sequential system with a chemically defined medium in porcine IVC as a routine approach [16,21], while others have used a sequential system with a natural mixture of oviductal (OF) and uterine fluids (UF) (Natur-IVF) [22].

Recently, extracellular vesicles (EVs), have been proposed as key components of oviductal secretions and as potential modulators of the maternal–gamete/embryo cross-talk in different species [23,24,25]. EVs are membrane-enclosed vesicles loaded with diverse molecular components (mRNAs, small non-coding RNAs, proteins, lipids, and metabolites) [26]. Oviductal EVs (oEVs) present in the oviductal fluid might act as natural nanoshuttles, bringing key components from the oviduct into gametes and embryos and in this way playing important roles in sperm functions [27,28,29,30] and early embryos [31,32,33]. In porcine, recent studies from our laboratory have shown that the porcine oEVs (poEVs) interact with both the spermatozoa and cumulus–oocyte complex, delivering OVGP1 into the ooplasm and increasing sperm survival [30]. Moreover, the poEV supplementation in IVF media has improved monospermic penetration during IVF [29] and point at the oEVs as being components of OF that are responsible for regulating polyspermy fertilization. So far, no studies have evaluated the effect of poEVs during IVC as potential boosters of embryonic development in porcine IVP systems. In bovine, oEVs during IVC have enhanced embryo development and cryosurvival [31,32] and induced alterations in the embryonic transcriptome [33].

Therefore, the aim of the present study was to determine the effect of poEVs on embryo development and their impact on the embryonic transcriptome. Besides this, the effect of poEVs was compared with complete OF on embryo development by using an in vitro sequential system (2 days versus 7 days of IVC), considering the importance of mimicking the maternal environment and with the idea of using a unique strategy to improve porcine IVP. Additionally, an integrative analysis of poEV cargo and embryonic transcriptome datasets was performed to unveil the potential of poEVs as modulators of the embryonic transcriptome.

## 2. Materials and Methods

### 2.1. Experimental Design

**Figure 1:**
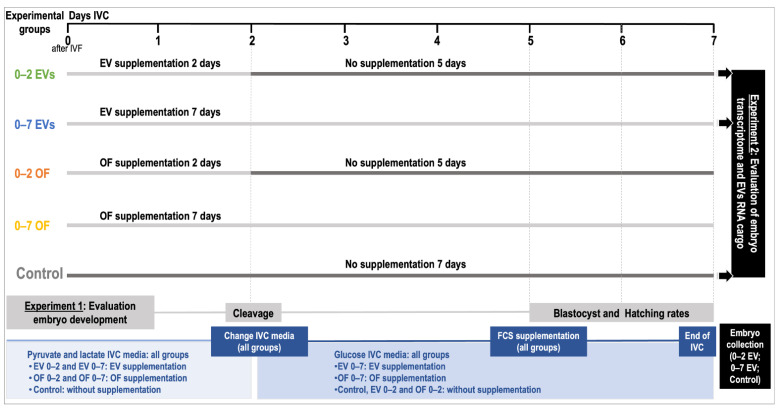
Schematic representation of the experimental design.

In Experiment 1, the effect of oEVs and complete OF in an IVC sequential system vs. non-sequential system on embryo development was examined. Since porcine embryos remain only two days in the oviduct from zygote to the 4-cell stage, at which stage they migrate to the uterus, oEVs as well as OF supplementation were used in IVC media for 2 days (0–2) followed by 5 days of IVC in fresh medium with no supplementation until day 7 of embryo development (sequential system) (Day 0 = day of fertilization). The effect of oEVs and OF for 7 days in IVC (0–7) was also tested (non-sequential system). In total, 5 different IVC groups were used: (1) OF 0–2 days; (2) OF 0–7 days; (3) EVs 0–2 days; (4) EVs 0–7 days; (5) control (non-supplementation). The selection of the concentrations for oEVs (0.2 mg protein/mL) and OF (10%) were based on previous studies in porcine and bovine [29,30,32,34] carried out in our laboratory and adapted to practical application in porcine IVC. So far, the physiological concentrations of oEVs in the oviduct are unknown. Furthermore, to avoid variability among oEVs and OF additives, the same pool of oviducts was used as a source for OF and oEVs. In Experiment 2, IVP embryos derived from EV 0–2, EV 0–7, treatments, and control embryos, as well as poEVs, were analyzed by RNA-sequencing (RNA-seq). Figure 1 illustrates a schematic representation of the experimental design.

### 2.2. Culture Media

Unless otherwise indicated, all chemicals were purchased from Sigma Chemical Co. (Saint Louis, MO, USA).

The medium used for the collection and washing of cumulus–oocyte complexes (COCs) was HEPES-buffered tissue culture medium 199 (TCM199-H; M7528) supplemented with 4 mg/mL gentamicin (G1272) and 1 mg/mL BSA. The oocyte IVM medium was TCM199 (M4530) supplemented with 10 IU/mL eCG (Syncro-Part, CEVA Santé Animal, Libourne, France), 10 IU/mL hCG (Chorulon, MSD Santé Animal, Beaucouzé, France), 570 mM cysteamine (M9768), 10 ng/mL epidermal growth factor (EGF; E4127), 10% fetal calf serum (FCS; F9665), 0.21 mM penicillin G and 0.07 mM streptomycin sulphate. The basic medium used for fertilization was essentially the same as that used by Abeydeera and Day [35]. This medium, designated as modified Tris-buffered medium (mTBM), consisted of 113.1 mM NaCl, 3 mM KCl, 7.5 mM CaCl_2_·2H_2_O, 20 mM Tris (crystallized free base), 11 mM glucose, 5 mM sodium pyruvate and was supplemented with 2 mM caffeine and 0.2% BSA (fraction V; A 7888, initial fractionation by cold alcohol precipitation). The IVC was a sequential medium based on NCSU-23 supplemented with 0.4% BSA [36].

### 2.3. Oviductal Fluid Collection

Gilt oviducts were collected from a local slaughterhouse and transported to the laboratory in a container maintained at 34–38 °C. The estrus cycle stage was assessed on the basis of ovarian morphology as previously described [37,38]. Oviducts from females at the pre-ovulatory stage of their estrus cycle (more than four 0.8–1.0 cm red follicles on each ovary) were selected (n = 12) and each oviduct was flushed from the ampulla to isthmus with 500 µL of PBS (P4417-TAB). Oviducts were gently pressed from the ampulla to isthmus while flushing in order to maximize fluid recovery. Both oviducts’ flushing (OF sample) from the same female were pooled and centrifuged at 300× *g* for 15 min to remove the cells. The supernatant was transferred to a new tube and centrifuged at 12,000× *g* for 15 min at 4 °C to remove cellular debris and apoptotic bodies. Clarified OF samples were stored at −80 °C until EV isolation. The same sources of OF samples were used for EV isolation to avoid variability. A pool of oviducts from 4 animals was used for each OF sample.

### 2.4. Isolation of Oviductal Extracellular Vesicles

Oviductal EVs were isolated from clarified OF (pool of four animals per replicate; in four replicates) by serial ultracentrifugation [39] and following the same protocol as previously described in [29,30,40]. Briefly, clarified OF was prepared by serial centrifugations, 300× *g* 15 min, 2000× *g* 15 min, and 12,000× *g* 30 min at 4 °C. Then, the supernatant was centrifuged at 100,000× *g* for 90 min at 4 °C. Then, the supernatant was removed, and the pellet was resuspended with 4 mL of PBS and centrifuged again at 100,000× *g* for 90 min at 4 °C. The final pellet was resuspended with 75 µL of NSCU-23 medium, aliquoted and stored at −80 °C. An aliquot of oviductal EVs and OF was used to determine the protein concentration by a bicinchoninic acid assay (BCA; Interchim, Montluçon, France) according to the manufacturer’s instructions using BSA as a standard. The day of the experiment, oviductal EVs samples were thawed, diluted in IVC media, filtered (0.22 µm) and used for preparing embryo IVC droplets.

### 2.5. Transmission Electron Microscopy (TEM)

To confirm the presence of oEVs in the oviductal fluid, TEM was used as previously described by [29,40]. Briefly, a 3 µL aliquot of the vesicle suspension was placed on a Formvar–carbon-coated grid and incubated for 5 min at room temperature in a moist chamber. Samples were washed with distilled water 3 times for 10 s each time by applying a drop of ten microliters of water. After each washing, a drop of water was removed by the filter paper touching the edge of the grid; a drop of 10 µL of 4% uranyl acetate water solution was applied to the grid three times for 20 s each time. A drop of solution was removed by the filter paper touching the edge of the grid. After removing the last uranyl acetate solution drop, the grid with the sample was dried in air at room temperature. Micrographs were obtained using an Hitachi TEM (HITACHI HT 7700 Elexience, Tokyo, Japan) at 80 kV (with a charge-coupled device (CCD) camera) and the JEM 1011 (JEOL, Tokyo, Japan) TEM equipped with a Gatan digital camera driven by Digital Micrograph software (Gatan, Pleasanton, CA, USA) at 100 kV. Image processing and the analysis of the size distribution of the EV population was performed using ImageJ software (National Institutes of Health, Bethesda, Rockville, MD, USA).

### 2.6. Porcine Embryo In Vitro Production

#### 2.6.1. Oocyte Collection and In Vitro Maturation (IVM)

Ovaries were obtained from a local slaughterhouse and transported to the laboratory in 0.9% NaCl at 37–39 °C. COCs were collected from antral follicles (3–6 mm diameter) in a 50 mL Falcon tube through an 18 g needle connected to a controlled vacuum system (30 mm Hg). Only COCs with a homogeneous ooplasm and surrounded by at least three complete and compact cumulus cells layer were selected. Then, COCs were washed twice in TCM199-H and subsequently washed three times in IVM medium before placing them in final IVM wells. Groups of 80 COCs were matured at 38.8 °C in a 5% CO_2_ atmosphere with maximum humidity in 500 µL of IVM medium under mineral oil for 22 h with hCG and eCG and for another 22 h in the same medium without hormones.

#### 2.6.2. Sperm Preparation and In Vitro Fertilization (IVF)

Frozen semen from a pool of two Large White boars were used [41]. For each IVP replicate performed, two straws of semen were thawed in a water bath at 38 °C for 20 s, their content was gently deposited on the discontinuous Percoll gradient (45–90% *v*/*v*; Pharmacia, Uppsala, Sweden) and was centrifuged for 20 min at 700× *g*. The selected spermatozoa were washed by centrifugation in IVF medium at 100× *g* for 10 min. The resulting pellet was resuspended to a final concentration of 1 *×* 10^6^ cells/mL with IVF medium. In vitro matured COCs were washed three times in IVF medium. Groups of 40 oocytes were co-incubated with 0.5 *×* 106 spermatozoa/mL in 100 µL drops under mineral oil for 16 h at 38.8 °C in a 5% CO_2_-humidified atmosphere.

#### 2.6.3. In Vitro Embryo Culture

Presumptive zygotes were removed from the fertilization medium and washed three times in pre-equilibrated IVC medium. Subsequently, they were transferred to a 4-well multidish (~30 zygotes per well), with each well containing 50 µL of IVC medium with different supplementation according to the experimental design and under mineral oil. Embryos were cultured at 38.8 °C in 5% CO_2_ in air for 18 h to assess the fertilization parameters or for 7 days to assess embryo development. Presumptive zygotes used for embryo development assessment from all groups were cultured for the first 2 days in glucose-free NCSU-23 supplemented with 0.33 mM pyruvate and 4.5 mM lactate. Following 48 h of culture, all presumptive zygotes were removed and cultured subsequently in fresh NCSU-23 medium containing 5.5 mM glucose until day 7. On day 5 of embryo development, IVC media were supplemented with 10% FCS (Biomedicals, Santa Ana, CA, USA; MP-5418).

#### 2.6.4. Assessment of In Vitro Fertilization Parameters

To evaluate fertilization parameters, presumptive zygotes from the different treatments were fixed on slides with acid acetic/ethanol solution (1:3 *v*/*v*) for at least 48 h, washed with absolute ethanol for 10 min and stained with 1% lacmoid (*w*/*v*; 274720). The sperm penetration and pronuclear formation were evaluated by phase-contrast microscopy at 400× magnification. Oocytes were considered to be penetrated when they had one or more swollen sperm heads and/or male pronuclei, with their corresponding sperm tails present, and two polar bodies. Oocytes with an abnormal appearance or that were degenerated or immature were not counted. The fertilization parameters evaluated were penetration (number of oocytes penetrated/total inseminated), monospermy (number of oocytes containing only one male pronucleus/total penetrated) and the efficiency of fertilization (number of monospermic oocytes/total inseminated).

#### 2.6.5. Assessment of Embryo Development and Embryo Quality

To examine the ability of embryos to develop to the blastocyst stage in vitro, presumptive zygotes were in vitro cultured for 7 days under the different treatments. On day 2, cleavage rate (number of embryos cleaved/total in culture × 100) and on day 5, day 6 and day 7, blastocyst rate (number of blastocyst/total in culture × 100) was evaluated under a stereomicroscope. Embryo development was evaluated at different days (5–7) to examine if the use of different treatments affected the kinetics of development. An embryo which had two or more cells was counted as cleaved and an embryo with a clear blastocele was defined as a blastocyst. A blastocyst with a broken or lost zona pellucida (ZP) was considered as a hatching or hatched blastocyst (hatching rate calculated as the ratio of hatching or hatched blastocysts to the total number of embryos or blastocyst evaluated × 100). The total cell number, as an indicator of embryo quality, was evaluated by fixing the embryos in 4% paraformaldehyde for 30 min, washing them twice with PBS supplemented with 3 mg/mL BSA, staining them with 10 µg/mL Hoechst 33342 for 15 min and mounting each blastocyst in 4 µL of Vectashield (Vector, Burlingame, CA, USA), followed by examination using a fluorescence microscope.

### 2.7. Statistical Analysis

Cleavage (day 2), blastocyst (day 5–7) and hatching rates (day 7), as well as the number of cells/blastocysts are presented as mean ± SEM. All variables were previously tested for their normality (Shapiro–Wilk test). Variables following normal distribution were analyzed by a one-way analysis of variance (ANOVA) followed by the Tukey’s test for multiple comparisons. Variables not following a normal distribution were analyzed by the Kruskal–Wallis test followed by Dunn’s test for multiple comparisons. For all variables *p* < 0.05 was considered statistically significant.

### 2.8. Transcriptomic Analysis of poEV Cargo and Embryos by RNA-Sequencing: RNA Isolation, Low-Input Total RNA Library Preparation, Sequencing and Data Analysis

A total of five different poEV samples (n = 5), each resulting from a pool of three oviducts, were used for RNA-sequencing. For the embryos, four replicates from each embryo group (EV 0–2, EV 0–7 treatments and control) collected at the blastocyst stage were used for transcriptomic analysis by RNA-sequencing (RNA-seq). Each replicate represented a pool of 3 embryos at the blastocyst stage, which were washed and snap-frozen in liquid nitrogen on day 7 of development and used for RNA isolation.

The total RNA from these 12 embryo pool samples and 5 poEV samples was isolated using the miRNeasy micro kit (QIAGEN AG, Hombrechtikon, Switzerland) according to the manufacturer’s instructions. RNA quality and concentration were analyzed using the Agilent 2100 Bioanalyzer (Agilent Technologies, Santa Clara, CA, USA) and NanoDrop (Thermo Fisher, Waltham, MA, USA). The three replicates per embryo group and the five poEV samples with the best RNA quality and concentration were selected for preparation of RNA-seq libraries (Average RNA integrity number (RIN) EV samples: 7.7; Average RIN Embryos: 6.7). In total, 14 libraries were prepared: 9 libraries for embryos; 3 replicates/embryo treatment; 5 libraries for poEVs.

RNA-Seq library preparation was performed by using the SEQuoia Complete Stranded RNA Library Prep Kit (Bio-Rad Laboratories, Inc. Cressier, Switzerland), which permits the capture of long as well as short RNAs in a single library. For poEV samples, 200 ng total RNA was used and for embryos, samples of 1 ng were used. One pool of 14 samples (5 poEVs samples and 9 embryo samples, 3 replicates/embryo group) was prepared, and sequencing was performed on one SP flow cell on an Illumina NovaSeq 6000 instrument (Functional Genomic Center Zurich). Paired-end sequencing was performed with 92 bp for read one (cDNA insert) and 8 bp for read 2 (UMI sequence for removal of PCR duplicates).

Data analysis was performed in a locally installed version of Galaxy [42]. Sequencing reads were processed using Cutadapt (Galaxy version 1.16.8) with the parameters -u 1 (trim first base at 5′), -a A{10} (trim any poly(A) track and following bases in the read), -m 15 (removes reads shorter than 15 bases), and a quality cutoff of 28. Trimmed reads were mapped to the current porcine genome reference assembly (Sscrofa11.1) with HISAT2 (Galaxy version 2.1.0 + galaxy4). NuDUP mark/remove PCR duplicates based on molecular tags (Galaxy version 2.3.3) was used to remove PCR duplicates from the BAM files before counting reads mapped to the annotated features of the porcine genome with the tool featureCounts (Galaxy version 1.6.4 + galaxy1). The latest NCBI GFF3 genome annotation file (GCF_000003025.6_Sscrofa11.1) was used. A separate counting was performed for reads mapping to mature microRNAs (miRNAs) with the MiRDeep2 Quantifier (Galaxy version 2.0.0) based on porcine, bovine, and human miRNA sequences of miRBase (version 22.1). MicroRNAs that showed at least 10 counts in at least 2 out of 3 samples for embryo treatments were used for further differential expression analysis.

Further analysis was performed in R with the BioConductor package TCC [43] to identify differentially expressed genes (DEGs) and miRNAs. In TCC, the parameter norm.method = “tmm” was used for normalization and test.method = “edger” for differential gene expression analysis (multiclass design) was used [44]. Genes as well as miRNAs with *p*-values < 0.01 were selected as DEGs between the following comparisons: EV 0–2 vs. control (E2C); EV 0–7 vs. control (E7C); EV 0–2 vs. EV 0–7 (E7_2). A false discovery rate (FDR) <10% was also considered for highly significant DEGs. Functional annotation analysis was performed using Metascape online tool (www.metascape.org, accessed on 19 July 2022) [45]. A microRNA target analysis was performed with the MIENTURNET webtool (database miRTarBase) (http://userver.bio.uniroma1.it/apps/mienturnet/, accessed on 19 May 2022) [46] and with DIANA tools MirPath v.3 (https://dianalab.e-ce.uth.gr/html/mirpathv3/index.php?r=mirpath, accessed on 3 May 2022) [47]. To identify enriched functional terms for target genes of miRNAs identified in embryos, DAVID functional annotation clustering was used (https://david.ncifcrf.gov/, accessed on 19 May 2022) [48].

### 2.9. Quantitative Real-Time Polymerase Chain Reaction (qPCR) Analysis

Embryonic gene expression analysis for 6 selected genes, based on RNA-seq results, was performed in EV 0–2 vs. control and EV 0–7 vs. controls by qPCR. The genes analyzed were: S100 calcium-binding protein A11 (*S100A11*), annexin A2 (*ANXA2*), GABA type A receptor-associated protein-like 1 (*GABARAPL1*), heat shock protein A5 (*HSPA5*), tryptophanyl–tRNA synthetase 1 (*WARS*) and Sirtuin 1 (*SIRT1*). The primer sequences for these genes are listed in Table 1. First, Crescendo cDNA synthesis for the qPCR kit (TECAN Sales Switzerland AG, Maennedorf, Switzerland) was used to generate amplified cDNA from the total RNA of embryo samples. The same RNA samples used for RNA-seq were used (0.5 ng total RNA). Subsequently, the mRNA expression of the selected genes was measured in the amplified cDNA by qPCR on a Light Cycler 96 (Roche Diagnostics (Schweiz) AG, Rotkreuz, Switzerland) with the KAPA HiFi HotStart PCR Kit (Roche Diagnostics (Schweiz) AG, Rotkreuz, Switzerland), adding EvaGreen^®^ Dye, 20× in water (Biotium). The qPCR was performed in a reaction volume of 20 μL, consisting of 4 μL 5× Kapa HiFi Buffer, 0.6 μL Kapa dNTP mix (10 mmol/L), 0.4 μL Kapa HiFi HotStart DNA polymerase, 0.6 μL of each primer (10 μmol/L), 1 μL Eva Green Dye, 11.8 μL water and 1 μL cDNA template. Cycle parameters of the PCR were 95 °C for 3 min, followed by 45 cycles of 98 °C for 20 s, a specific annealing temperature for 15 s and 72 °C for 15 s, and then a melting step (95 °C for 10 s, 65 °C for 60 s and 97 °C for 1 s). Melting curves of the amplified PCR products were obtained for confirmation of specific amplification. A no-template control (RNA sample) was included for each primer pair. The Cq values (quantification cycle) determined for the selected genes were normalized against the geometric mean of three reference genes, 18S rRNA (*RNS18*), ubiquitin B (*UBB*) and H3.3 histone A (*H3F3A*). Relative expression differences between EV 0–2 vs. control and EV 0–7 vs. control were calculated, and a *t*-test was performed in Microsoft Excel. *p*-values <  0.05 were considered significant.

## 3. Results

### 3.1. Transmission Electron Microscopy of Oviductal Extracellular Vesicle Preparations

**Figure 2:**
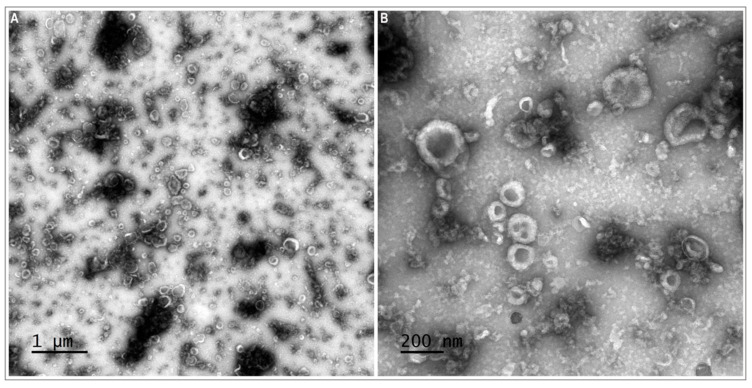
Transmission electron microscopy (TEM) images of porcine oviduct extracellular vesicles (oEVs). Representative wide-field (**A**) and close-up images (**B**) of extracellular vesicles, (small vesicles 30–100 nm in size and larger vesicles > 100 nm in size).

TEM observations confirmed the presence of EVs in the oviductal fluid. TEM preparations showed a population of small EVs (30–100 nm) resembling exosomes and a small population of larger EVs (>100 and up to 1000 nm) resembling microvesicles (Figure 2).

### 3.2. Protein Concentration of Oviductal Secretions Used as Supplements in In Vitro Embryo Culture

A total of three pooled samples of OF and three oEV pooled samples derived from the same animals were collected and used as supplements during in vitro embryo development. To examine the variability among pools for OF as well as oEVs, the protein concentration of all three pools of EVs and OF samples was analyzed. Protein concentrations (mg/mL) for EVs samples were 21.8, 24.2 and 28.3 mg/mL for Pool 1, Pool 2 and Pool 3, respectively. Protein concentrations (mg/mL) for OF were 7.38, 9.64 and 12.07 mg/mL for Pool 1, Pool 2 and Pool 3, respectively. These three pools were used in the six replicates of IVP.

### 3.3. In Vitro Embryo Production with Different Oviductal Secretions during In Vitro Culture

A total of 1232 oocytes were matured, fertilized, and cultured in vitro in this study in six different replicates (125–256 oocytes/replicate). From these, 139 presumptive zygotes were used for evaluating IVF parameters after 16 h of in vitro culture. The remaining zygotes were cultured in vitro for 7 days under different treatments (210–220 presumptive zygotes/treatment group). Overall, good maturation rates were obtained for all replicates (85.6% ± 1.3), with high penetration rates (79.9% ± 0.7), but with low monospermy rates (27% ± 1.0), giving a total efficiency of the IVF system of around 22% (21.6% ± 0.9).

**Figure 3:**
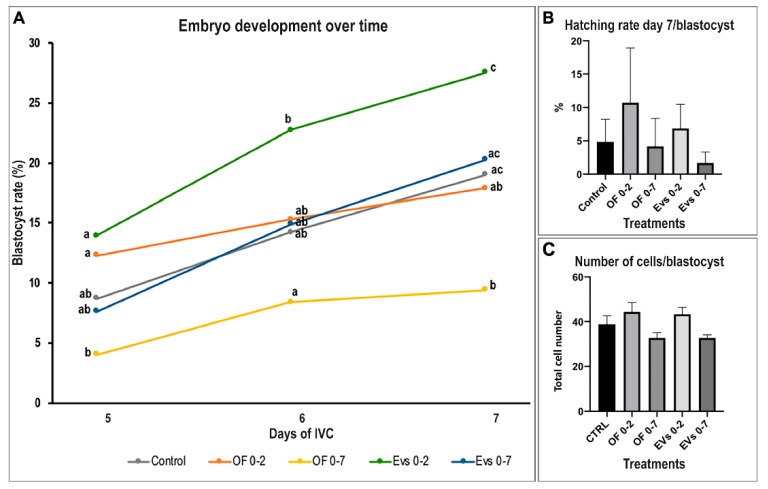
Effect of porcine oviduct extracellular vesicles (oEVs) and oviductal fluid (OF) supplementation in a sequential and non-sequential in vitro culture system on embryo development and quality over time (Mean ± SEM). (**A**) Graph shows blastocyst rate (%), with different letters indicating significant differences within groups at one specific time point of development (day 5–7) (*p* < 0.05). (**B**) Graph illustrates the hatching rate/blastocyst, representing the percentage of embryos that were hatching or completely hatched on day 7 of IVC/total embryos reaching the blastocyst stage on day 7. (**C**) Graph shows number of cells/blastocyst.

Regarding the effect on embryo development, the use of EV 0–2 treatment significantly increased the cleavage rate when compared to the control (51.8% ± 2.1 EV 0–2 vs. 44.9% ± 0.8 control; *p* < 0.05) (Table 2). Figure 3 shows the effect of supplementation with different oviductal secretions on the blastocyst rate over time, showing a clear enhancement of embryo development when EV 0–2 treatment was used compared to the other treatments. Particularly, EVs 0–2 supplementation showed a significant increase in the blastocyst rate on day 7 when compared to both OF treatments (*p* < 0.05). Interestingly, the use of OF 0–7 significantly decreased blastocyst yield (*p* < 0.05) but not EVs 0–7, which was similar to control. Regarding embryo quality, number of cells/blastocysts showed a tendency to increase for EVs 0–2 and OF 0–2 when compared to the longer-culture EV treatment (EV 0–2 vs. EV 0–7 *p* = 0.092; OF 0–2 vs. EV 0–7 *p* = 0.194) (Figure 3).

### 3.4. RNA Cargo of poEVs

RNAs of a total of 12,998 different genes and 163 miRNAs were identified in poEVs. Around 30% of RNAs identified in poEVs were found with >500 read counts per transcript (3804), 15% (1914) were found with >1000 reads, and 1% were found with >10,000 reads (115). For the miRNAs, around 30% (50) of miRNAs identified in poEVs were found with >100 counts, 11% (18) with >500 counts, and 6% (10) with >1000 counts. A detailed list of RNAs and miRNAs identified in poEVs can be found in Supplementary Data S1, Tables S1 and S2.

DAVID functional annotation clustering for RNAs with >1000 reads (1914) revealed enrichment in GO terms related to RNA binding (enrichment score (ES): 57.75); cytoplasm translation (44.64); extracellular exosome, extracellular vesicle (27.39); cytoskeleton organization (13.93); programmed cell death (10.97); response to stress (10.95); regulation of cell communication (6.62); interferon signalling (5.76); response to oxidative stress (5.31); apoptosis (4.69); response to carbohydrates (3.08); response to nutrient levels (2.95); reproductive processes (2.88); cell proliferation (2.84); regulation of growth (2.77), embryo development (2.1) (GO terms: in utero embryonic development; embryo development ending in birth or egg hatching; embryo development and chordate embryonic development); regulation of gene silencing by miRNA (2.0) (Supplementary Data S1, Table S3).

Predicted target analysis for all miRNAs identified in poEVs (163) (with 132 miRNAs out of 163 found in MIENTURNET) provided 9702 and 12,692 predicted target genes based on TargetScan and miRTarBase, respectively (Supplementary Data S1, Tables S4 and S5). Focusing on the 50 miRNAs more abundant in poEVs (>100 reads) provided 4907 and 7180 predicted target genes based on miRTarBase (FDR 0.5) and Target Scan (FDR 0.5), respectively (Supplementary Data S1, Table S6). An overlap of 2476 target genes was obtained from both databases, which was used for further functional analysis by DAVID. Functional annotation clustering revealed enriched GO terms associated with the regulation of metabolic process (ES: 23.83); regulation of cell communication (21.07); apoptotic process (19.2); cellular protein modification process (19.1); cellular response to stress (15.9); regulation of cell differentiation (15.11); cell proliferation (14.23); embryonic morphogenesis (11.06); embryo development (7.35) (GO terms: embryo development ending in birth or egg hatching; in utero embryonic development); regulation of gene silencing by miRNA (4.4); response to oxidative stress (2.19) (Supplementary Data S1, Table S7).

### 3.5. Transcriptome Profile of EVs Treated and Control Embryos

#### 3.5.1. Embryonic RNA Profiles

A total of 2603 RNAs and 34 miRNAs were identified across all porcine embryo samples regardless of the treatment (Supplementary Data S1, Tables S8 and S9). Principal component analysis (PCA) based on all identified miRNAs revealed a separation of treated and control embryos in principal component (PC) 1 (Supplementary Figure S1, each sample label refers to: number of embryo pools for each treatment (1–3; 5,6 and 8; 9–10 and 12) and the treatment used (EV 0–2: 0_2; EV 0–7: 0_7 or CO; replicate (R1, R2 and R4)). EV 0–2 and EV 0–7 samples seemed also to be separated based on PC2. Similarly, a PCA on all identified RNAs was also performed, but the three embryo groups did not show a clear separation (Supplementary Figure S1).

**Figure 4:**
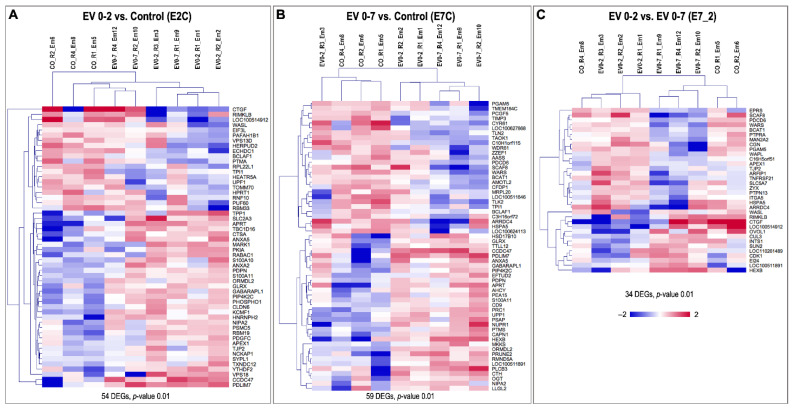
Unsupervised hierarchical clustering (HCL) of differentially expressed genes (DEGs). (**A**) HCL of the 54 DEGs (*p*-value: 0.01) confirmed a clustering of the samples according to treatment EV 0–2 vs. control (E2C). (**B**) HCL of the 59 DEGs (*p*-value: 0.01) confirmed a clustering of the samples according to treatment EV 0–7 vs. control (E2C). (**C**) HCL of the 34 DEGs (*p*-value: 0.01) confirmed a clustering of the samples according to treatment EV 0–2 vs. EV 0–7 (E7_2). HCL was performed with Pearson correlation coefficient by MeV software (Multiple Experiment Viewer, MeV v.4.8.1, https://sourceforge.net/projects/mev-tm4/, accessed on 19 May 2022) [49]. Rows indicate DEG, while columns represent embryo samples under different treatments. Mean-centered expression values (log2 counts per million of a sample − mean of log2 counts per million of all samples) for all embryo samples are shown. Color scale is from −2 (blue, lower than mean) to 2 (red, higher than mean).

Differential gene expression provided 54, 59 and 34 DEGs (based on *p*-value < 0.01) in EV 0–2 vs. CO (E2C), EV 0–7 vs. CO (E7C), and EV 0–7 vs. EV 0–2 (E7_2) comparisons, respectively (for FDR < 0.1; 2, 4 and no DEGs were found, respectively) (Figure 4). In E2C, 34 (63%) genes were upregulated vs. 20 (37%) downregulated. In E7C, 32 (54%) genes were upregulated vs. 27 (46%) downregulated. In E7_2, 13 (38%) genes were upregulated vs. 21 (62%) downregulated. A complete list of DEGs for each comparison can be found in Supplementary Data S1, Tables S10–S12.

**Figure 5:**
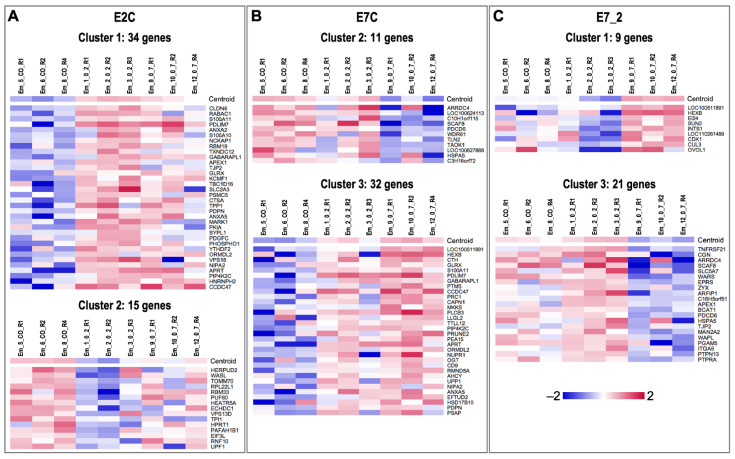
Clustering of DEGs with similar expression profiles across embryo treatments. SOTA clustering (Multiple Experiment Viewer, MeV v.4.8.1, https://sourceforge.net/projects/mev-tm4/, accessed on 19 July 2022) [49] resulted in 3 clusters with similar expression profiles. Mean-centered expression values (log2 counts per million of a sample − mean of log2 counts per million of all samples) are shown. Color scale is from −2 (blue, lower than mean) to 2 (red, higher than mean).

Additionally, SOTA clustering analysis was performed for the DEGs of each comparison, revealing three clusters of genes with similar expression profiles for the comparisons E2C, E7C, and E7_2 (Figure 5). For E2C, Cluster 1 contained 34 genes that were downregulated in CO compared to EV 0–2 (Figure 5A). Cluster 2 had 15 genes upregulated in CO compared to EV 0–2 (Figure 5A). Cluster 3 (five genes) did not show a clear pattern (not shown). For E7C, Cluster 2 contained 11 genes downregulated in EV 0–7 compared to CO (Figure 5B). Genes in Cluster 3 (32) were upregulated in EV 0–7 compared to CO (Figure 5B). Genes in Cluster 1 (16) did not show a very homogeneous pattern regarding the three groups (not shown). For the comparison E7_2, the nine genes of Cluster 1 were downregulated in EV 0–2 compared to EV 0–7 (Figure 5C). Cluster 3 contained 21 genes with increased expression in EV 0–7 compared to EV 0–2 (Figure 5C). The four genes of Cluster 2 did not show a very clear pattern regarding the groups (not shown).

**Figure 6:**
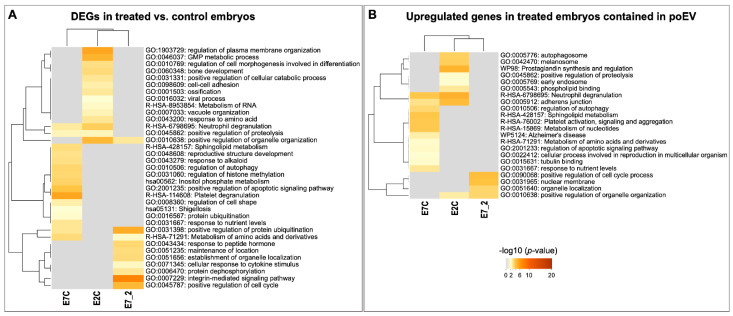
Heatmaps of overrepresented GO categories and molecular pathways of the DEGs derived from the three comparisons among embryo treatments. The heatmap cells are colored by their *p*-values from gray (not significant, lack of enrichment) to brown (highly significant). Image created with Metascape webtool (https://metascape.org, accessed on 19 July 2022) [44] and modified with Adobe Photoshop v.22.4.3 (Adobe, San Jose, CA, USA). (**A**) Heatmap of the DEGs derived from the three comparisons among embryo treatments. (**B**) Heatmap of the upregulated DEGs derived from the three comparisons among embryo treatments and contained in poEV.

Functional annotation analysis of the DEGs was performed with the Metascape online tool (https://metascape.org, accessed on 19 May 2022) [44]. Figure 6 illustrates enriched functional terms for each set of DEGs derived from each comparison, showing a unique set of GO terms for each comparison and a small overlap among comparisons. Specifically overrepresented terms for E2C were related to the positive regulation of organelle organization, GMP metabolic process, regulation of cell morphogenesis involved in cell differentiation, cell–cell adhesion, and response to amino acids. Terms specific for E7C were related to platelet degranulation, positive regulation of apoptotic signaling pathways, the regulation of cell shape, response to nutrients, and regulation of histone methylation. Unique terms for E7_2 were related to positive regulation of protein ubiquitination, integrin-mediated signaling pathways, the positive regulation of the cell cycle, metabolite of amino acids and derivatives, and response to peptide hormones (Supplementary Data S1, Table S13).

#### 3.5.2. Embryonic miRNA Profiles

Based on a *p*-value cut-off of <0.01 (FDR <0.1), six, seven, and three miRNAs were differentially expressed (DE) for E2C, E7C, and E7_2 comparisons, respectively (Supplementary Data S1, Tables S14–S16). The predicted targets for these DE miRNAs were identified using MIENTURNET. Because of the overlap of DE miRNAs among comparisons, 10 unique DE miRNAs were used for target gene analysis. Nine were found in the MIENTURNET webtool (not found: bta-miR-11972) and seven with functional enrichment analysis (Supplementary Data S1, Table S17). The list of DE miRNAs and their associated predicted target genes and GO terms and pathways can be found in Supplementary Data S1, Tables S18 and S19. For example, hsa-miR-302a-3p and hsa-miR-7-5p have been related to preimplantation embryos, targeting NANOG and KLF4, respectively. DE miRNAs were also involved in the estrogen signalling pathway, including hsa-miR-302a-3p, hsa-miR-302b-3p, and hsa-miR-302d-3p, targeting AKT1, while hsa-miR-7-5p targets BCL2 and FOS. Related to TGF-beta signaling pathway and signaling pathways regulating the pluripotency of stem cells were hsa-miR-1260b, hsa-miR-302a-3p, hsa-miR-302b-3p, hsa-miR-302d-3p, and hsa-miR-7-5p (Supplementary Data S1, Tables S20 and S21).

### 3.6. Integration of Embryo and poEVs Datasets

#### 3.6.1. Comparison of RNAs Identified in EVs-Treated Embryos and Contained in poEVs

Of the 2603 RNAs identified in embryos, 2502 RNAs were also contained in poEVs (Supplementary Data S2, Table S1). With respect to the genes upregulated in EV-treated embryos (E2C: 34; E7C: 32), 32 and 31 RNAs, respectively, were also present in poEVs. The comparison with the downregulated genes (E2C: 27; E7C: 21) revealed 26 and 19 genes, respectively, of which their RNA was present in poEVs. For the E7_2 comparison (13 up- and 21 downregulated genes) 10 and 19 were in common with RNAs contained in EVs, respectively. The detailed lists of these comparisons can be found in Supplementary Data S2, Tables S2–S5.

#### 3.6.2. Comparison of miRNAs Identified in EVs-Treated Embryos and Contained in poEVs

Of the 33 miRNAs found in embryos, 22 (66.7%) were also detected in poEVs (Supplementary Data S2, Table S6). Among these 22, 9 and 11 miRNAs were found with increased abundance in EV-treated embryos compared to controls (E2C and E7C, respectively), while 13 miRNAs were found with a higher abundance in EV embryos treated for two days rather than seven days (E7_2). From the nine, one miRNAs, miR-7-5p, was a DE miRNA in E2C. From the eleven, two were DE miRNAs (miR-30e-3p, miR-574-5p) in E7C. From the thirteen miRNAs, two were DE miRNAs (miR-11972; miR-30e-3p) in E7_2 (Supplementary Data S2, Table S6).

Of the six, seven, and three DE miRNAs in embryos (E2C, E7C, and E7_2), two, four, and three were identified also in poEVs, respectively (Supplementary Data S1, Table S17). Enriched GO terms and pathways associated with these DE miRNAs were, e.g., longevity regulating pathway, apoptosis, focal adhesion, estrogen signaling (E2C, miR-7-5p) and signaling pathways regulating the pluripotency of stem cells (E7C, miR-1260b) (Supplementary Data S1, Tables S19 and S20).

#### 3.6.3. Comparison of Predicted Target Genes of miRNAs Identified in poEVs with RNAs in Embryos

Since MIENTURNET MirTarbase (12692) and TargetScan (9703) provided a different set of target genes for miRNAs in poEVs (Supplementary Data S1, Tables S4 and S5), both were compared to the 2603 RNAs identified in embryos, resulting in 2125 and 1626 common genes (Supplementary Data S2, Table S7). The overlap of both comparisons resulted in 1523 predicted target genes (Supplementary Data S2, Table S7). From these 1523 predicted target genes, 34, 29, and 20 were DE in E2C, E7C, and E7_2, respectively (Supplementary Data S2, Table S8).

These 1523 predicted target genes were also compared to genes downregulated in embryos in the three comparisons, resulting in 15, 14, and 14 genes for E2C, E7C, and E7_2, respectively (Supplementary Data S2, Table S9). The comparison to upregulated genes revealed 19, 15, and 6 genes for E2C, E7C, and E7_2, respectively (Supplementary Data S2, Table S9). The set of miRNAs potentially targeting the genes downregulated in E2C, E7C, and E7_2 is listed in Supplementary Data S2, Table S10.

On the other side, target genes were also obtained for the set of miRNAs, with a higher abundance in EV 0–2 embryos (17 miRNAs) and EV 0–7 embryos (17 miRNAs) compared to those present in the control and poEVs (23 unique miRNAs between both). This revealed 2906 predicted target genes (15 miRNAs out of 23 found in MIENTURNET; FDR 0.3) (Supplementary Data S2, Table S11). The DAVID functional analysis of these predicted target genes showed enriched GO terms related to apoptotic processes and cell death (ES: 18), cellular response to stress (ES:16.5), as well as with several GO terms linked to embryo development (ES: 4.64; with more than 200 predicted target genes) (Supplementary Data S2, Tables S12 and S13).

#### 3.6.4. Comparison of Porcine and Bovine oEV RNA Cargo and Their Potential Transcriptomic Alterations in Embryos

A comparison of porcine oEV RNA cargo to bovine oEVs from our previous study [39] revealed an overlap of 74.3% (9662) of RNAs and 32% (52) of miRNAs between studies or species. When the comparison was based on all the RNAs identified in embryos cultured with oEV in porcine vs. bovine, more than 90% (2361) RNAs were found to be in common. Among these common genes we found that 51 out of 54 DEGs in E2C and 50 out of 59 DEGs in E7C were also detected in bovine embryos cultures with EVs (7 days). When the comparison was focused only on the DEGs identified in porcine and bovine, only one gene (PDLIM7) was found as DE in EV-treated porcine and bovine embryos. The detailed lists can be found in Supplementary Data S2, Table S14.

#### 3.6.5. Validation of Embryonic Gene Expression Results and EV Transcript Abundance by qPCR of Selected Candidate Genes

Gene expression results by qPCR for five selected genes found by DEG in EV 0–2 vs. control or/and EV 0–7 vs. control embryos are illustrated in Table 3. Results of all selected genes by qPCR confirmed the RNA-seq data, although data for HSPA5 and GABARAPL1 were not statistically significant but showed a tendency (*p* = 0.0537 and 0.0936, respectively), as shown in Table 3. Moreover, the qPCR data demonstrated that SIRT1 together with HSPA5 and WARS, which could be targeted by different miRNAs contained in the porcine oEVs, were downregulated in EVs treated embryos (Table 3). Additionally, qPCR results showed the higher abundance of the selected transcripts in oEVs such as S100A11 and ANAX2, which correlated with RNA-seq data and with a higher gene expression in EV-treated embryos. By contrast, WARS and SIRT1, which were downregulated in EV-treated embryos (for EV 0–2 and EV 0–7, respectively), showed a lower abundance in oEVs (Table 3).

## 4. Discussion

The results of the present study showed that the use of poEVs, particularly during the first two days of IVC, enhanced embryo development. Besides, poEVs exerted a functional effect on embryos by modulating their transcriptome, given the different RNA and miRNA expression patterns obtained for EV-treated embryos compared to control embryos. Furthermore, differences were also found between two- and seven-day treatments, confirming the effect of the sequential system at the embryo transcriptomic level. In the next lines, the effects of poEVs compared to whole OF on embryo development, the importance of the sequential system and more extensively, the potential poEV components regulating the embryo transcriptome will be discussed.

### 4.1. Effect of poEVs on Embryo Development

The beneficial effect of poEV supplementation during two days of IVC on embryo development (EV 0–2) was reflected by an increased cleavage rate compared to the control, and with a similar trend for EV 0–7 embryos. A well-known phenomenon, called the “four-cell block”, occurs in porcine embryo developed in vitro at this time. This “block” is characterized by an arrest in cleavage at the four-cell stage and has been related to the maternal–zygotic transition of developmental control [50]. This “cell block” also occurs in other mammalian species, e.g., in the mouse (two-cell stage) [51], in bovine (eight-cell stage) [52] and in human (eight-cell stage) [53]. Many studies have shown that this developmental block can be overcome by the co-culture of embryos with oviductal or granulosa cells, supplementation with OF, as well as modifications of the composition of embryo culture media [36,54,55]. In a similar way, the poEV supplementation might bring important factors to the embryo and contribute to overcome the “four-cell block” and support the maternal–zygotic transition of developmental control. Moreover, EVs 0–2 treatment improved blastocyst yield over time, with clear results at day 7 of embryo development. The impact of the EVs 0–2 treatment on the embryo was reflected in the alteration of the embryo transcriptome with 54 DEGs, compared to the control. When EV 0–7 treatment was used, no significant differences were observed in blastocyst yield and quality compared to the control, but the effect of EVs 0–7 treatment was also reflected in an altered transcriptome, with 59 DEGs compared to the control. Overall, both poEVs treatments affected the embryonic transcriptome.

Similarly, Fang et al. [56] reported that oEV obtained from in vitro oviductal epithelial cell-conditioned media improved the development competence of parthenogenetic and somatic nuclear transfer porcine embryos. In contrast to our results, supplementation for 7 days provided better blastocyst rates than compared to three days with in vitro obtained oEVs. These authors also observed that nine genes (*BCL2*, *SOD1*, *NANOG*, *POU5F1*, *SOX2*, *GATA6*, *PNPLA2*, *LIPE*, *MGLL*) were upregulated and one was downregulated (BAX) after oEV treatment compared to control. However, none of these genes have been identified to be altered in our study, which could be due to the following: the effect was examined in parthenotes compared to embryos; the different oEV source (in vitro vs. in vivo) or the different technique used to analyze gene expression (qPCR vs. RNA-sequencing).

In bovine, we showed that the use of oEVs during the complete IVC enhanced blastocyst yield, quality, and embryo survival over time [32]. Besides, the oEV effect was reflected in the embryonic transcriptome changes, by altering the expression of around 200 genes in EV-treated embryos when compared to control embryos [33]. Lopera-Vasquez et al. [57] also observed an improvement of embryo cryosurvival when oEVs were used during complete IVC in bovine, although they did not find any increase in blastocyst yield. Comparative analyses of the transcriptome alterations between bovine and porcine have shown that most of the DEGs detected in E2C and E7C in our study were also identified as being expressed in bovine embryos, but only a few DEGs were shared between studies. The differences could be species-specific, but could also be due to the source of oEVs used, since the poEVs in the present study were collected from animals at the pre-ovulatory stage, while in bovine oEVs were obtained from animals at post-ovulatory stage [32]. The selection of the pre-ovulatory stage for this study is based on our previous studies, demonstrating that poEVs (pre-ovulatory stage) improve monospermy and modulate sperm motility and survival in porcine [29,30]. Besides, it relied on the idea of using a unique poEV strategy to improve all steps of the IVP and boosting the sperm-fertilizing ability as well as also embryo development. It is known that the oEVs’ molecular cargo is very dynamic and their mRNA, small RNA, protein and metabolite components change during the cycle [40,58]. The use of oEVs from post-ovulatory stages could bring about a higher increase in the blastocyst rate in porcine, accompanied by major alterations on the embryonic transcriptome as seen in bovine, since it would reflect more the physiological maternal environment, and calls for further studies. It is to be noted that differences between results of porcine and bovine studies could be also due to methodological aspects, e.g., RNA-seq library preparation, sequencing, and annotation. Overall, results from transcriptomic analyses in bovine and porcine embryos treated with oEVs together with the improvement of embryo development support the hypothesis that oEVs contain crucial factors contributing to successful embryo development. Besides, a comparison of the oEVs’ RNA cargo between porcine and bovine provided an overlap of 74% in RNA cargo and 32% at the miRNA level, pointing to common components that could contribute to embryo development and that are shared among species.

### 4.2. Effect of Sequential System and Oviductal Secretions

The different transcriptome profiles obtained for E2C and E7C also reflected the effect of the sequential system, which was further confirmed with the differences in the RNAs and miRNAs found in the E7_2 comparison. These findings brought up several questions regarding the timeframe of poEV uptake by the embryo and how long the effect on the embryo lasted: (i) whether the poEVs entered the embryo at the beginning of the culture or their uptake is over time (2 days vs. 7 days); (ii) when did the poEVs exert an effect on the embryo and did this effect last over time; (iii) in the case of a longer exposition, if there is an accumulative effect or a maintained effect over time. By answering these questions with further studies, we will know when to include poEVs in the IVP system to maximize positive effects.

The supplementation of oEVs was compared to OF in the present study in the IVC sequential system. Previously, by comparing the effect of poEVs and OF as supplements in IVF media, we showed that oEVs could be the components of the OF responsible for modulating polyspermy in porcine [29]. The increased blastocyst rates with poEVs compared to OF, mainly when a sequential media was used, pointed to poEVs as being key components of the OF, promoting embryo development. In this sense, embryotropic factors contained in OF could be taken up by the embryos via oEVs and exert a positive effect on supporting embryo development, while 10% OF supplementation might bring diluted free factors and EVs that did not show such beneficial effects on embryo development.

Canovas et al. [22] used a natural mixture of oviductal (OF) and uterine fluids (UF) (Natur-IVC) in a sequential system during porcine IVC, improving embryo quality (hatching rates and cell number/blastocyst) and resulting in embryonic gene expression and methylation patterns closer to in vivo produced blastocysts. In bovine, the use of OF for days 0–4 of IVC, mimicking the time of the bovine embryo in the oviduct, provided similar blastocyst rates and embryo cryotolerance to a sequential system with OF (0–4 days) followed by UF (4–9 days) [59]. Overall, these findings point to the importance of the maternal components present in OF that contribute to embryonic development and the impact of a sequential system on the in vitro embryo production.

Our results suggest that poEVs might be part of these beneficial components, representing a good strategy to use them in all steps of IVP. Although we have demonstrated that poEVs during IVF increased monospermy fertilization and improved blastocyst rates during IVC, this strategy might be contrary to the current trend of using chemically defined formulations in IVP systems. EVs represent ideal natural nanoshuttles that can bring a “cocktail” of the important in vivo molecules into the embryos [40,58] that are present in oviductal secretions but that are not available in current commercial and chemically defined IVC media. Our study identified specific RNA cargo which could contribute to the beneficial effects on the embryo. Further functional studies on these specific components of oEVs would open new avenues to design the following: (1) EVs uploaded with key synthetic components; (2) including them in new formulations for IVC media. In the light of our results in porcine and bovine, the use of oEVs could overcome the in vitro culture deficiencies and promote successful pregnancy.

### 4.3. EV RNA Cargo and Their Potential Impact on the Embryo

To identify the potential RNA cargo altering the embryonic transcriptome, an integrative analysis between poEV RNA cargo and embryonic transcriptome datasets was performed. To narrow down on potential RNAs with effects on the embryos, our approach focused on three likely situations: (1) poEV mRNAs could be transferred to the embryos, increase the concentration of these mRNAs in EVs-treated embryos, and eventually increase the expression of corresponding proteins; (2) in a similar way, miRNAs upregulated in EV-treated embryos derived from poEVs could exert an impact on the embryo; (3) based on the common mechanism of miRNA function, i.e., downregulating protein and mRNA expression, attention was given to downregulated RNAs in embryos, which could be targeted by poEV miRNAs. It is also known that miRNAs can indirectly upregulate cellular RNAs [60], but this effect has been less studied and therefore, it was not further investigated in this study.

### 4.4. Upregulated RNAs in Embryos with Potential poEVs Origin

A number of genes with an increased expression in EVs-treated embryos has been previously associated with interesting roles in reproductive processes. S100 calcium binding protein A11 (S100A11) has been shown to have an inhibitory action during IVF in mice [61]. In this respect, it has been suggested that S100A11 plays a role in sperm selection through its action on cumulus cells and to block polyspermy [61]. Likewise, S100A11 derived from poEVs could be involved in the decreased polyspermy rate observed in our previous study, where poEVs were added during IVF in porcine [29]. Hanaue et al. also showed that the S100A11 protein was expressed in oviductal epithelial cells from the ampulla and the isthmus but could not be detected in the OF [61]. Our study revealed that S100A11 mRNA is contained in the poEVs, and via EVs it might exert an effect on embryos, but so far, its potential effect on the embryo development has not been examined in detail. Annexin A2 (ANXA2) and ANXA5 have been identified as embryo-interacting proteins in the bovine oviduct [62] and were upregulated in EV-treated embryos. Particularly, ANXA2 is expressed at the apical surface of porcine oviductal epithelial cells and is the main sperm binding protein in pigs [63].

GABA type A receptor-associated protein-like 1 (GABARAPL1) has been associated with autophagy, regulating embryo survival. It was found to be upregulated in dormant blastocysts, suggesting an important role in extending the longevity of dormant blastocysts in utero during delayed implantation [64]. Glutaredoxin 2 (GLRX), which is part of the glutaredoxin system, provides cells with a reduced environment and protects against oxidative stress [65]. It has been suggested that the supplementation of small thiols during oocyte IVM may stimulate glutathione synthesis and enhance embryo development to the blastocyst stage [66], which is in line with our results.

### 4.5. Upregulated miRNAs in Embryos with a Potential poEVs Origin

More than 200 of the predicted target genes of miRNAs upregulated in treated embryos and also present in poEVs have been related to functions associated to embryo development. Here, we mention some of the identified upregulated miRNAs that have been identified in embryos previously or associated with reproductive roles. Mir-574-5p expression was upregulated in both E2C and E7C, and its expression has been also reported in human embryos [67]. In porcine, mir-574-5p has been detected in embryos and the endometrium on days 14 and 20 of pregnancy, respectively [68,69]. Mir-574-5p can target IL6, an inflammatory mediator, which was downregulated in E2C and E7C embryos, suggesting a potential way of the poEVs to modulate the embryo immunity. In this line, Barrancos et al. [70] identified the upregulation of miR-574-5p in the culture media of porcine endometrial explant after seminal plasma treatment, and associated this upregulation with an immune response. MiR-7-5p, upregulated only in E2C, has also been reported in the follicular fluid and its expression was altered in patients with polycystic ovary syndrome, compared to normal [71].

MiR-302 family members (miR-302a-3p, miR-302b-3p, miR-302d-3p) were among the miRNAs with increased expression in both E2C and E7C. MiR-302a-3p has been detected previously in EVs from human stem cells [72]. It has been associated with maintaining pluripotency and the regulation of cellular differentiation [72]. Besides, miR-302a and miR-302b were previously identified in the blastocoele of human blastocysts [73]. This together with the identification of these miRNAs in poEVs suggests that the expression detected in the embryos could have an embryonic origin but could be also transferred via poEVs to the embryo and play a role in maintaining the pluripotency of the cells.

### 4.6. Downregulated RNAs in Embryo Potentially Affected by miRNAs Derived from poEVs

Heat shock protein A5 (*HSPA5*), also known as glucose-regulated protein 78 kDa (*GRP78*), was identified to be downregulated in EV 0–7 vs. control embryos. *HSPA5* could be downregulated by miR-181a-5p and miR30a-5p contained in poEVs [74]. MiR-181a-5p has the ability to reduce endoplasmic reticulum (ER) stress through GRP78 [75,76]. *GRP78* has been referred as the “master regulator” of the unfolded protein response, a cell stress coping strategy used by the cells to recover homeostasis. Moreover, GRP78 has been shown to be essential for early embryo cell growth in mice [77]. *HSPA5* was expressed on the trophoblastic cell surface under stress and hypoxic conditions along with p53 [78]. We hypothesize that the downregulation of *HSPA5* in embryos potentially via poEV-derived miRNAs could help the embryos to cope with the stressful in vitro environment and improve their development when compared to controls.

Tryptophanyl-tRNA synthetase 1 (*WARS*) was also identified downregulated in EV 0–7 vs. control embryos and could be potentially targeted by four miRNAs contained in poEVs. *WARS* is essential for protein synthesis and acts as a cytokine in the inflammatory response [79]. It seems to facilitate TLR2 and TLR4 signaling to exert innate immune responses [80]. Additionally, it has been shown that *WARS* is actively released from injury sites in response to damage both in vitro and in vivo and then acts as a potent nonenzymatic cytokine that promotes the self-renewal, migratory, and differentiation capacities of endometrial stem cells to facilitate the repair of damaged tissues [81]. *WARS* downregulation through the miRNAs cluster: hsa-miR-99b/let-7e/miR-125a is involved in the regulation of pathogen recognition receptor-stimulated suppressive antigen-presenting cells [82]. So far, there is no information on *WARS* function and signaling in embryos. However, given the marked downregulation of the *WARS* in EV 0–7 compared to control embryos, we speculate that could be due to this miRNA cluster present in poEVs. In this way, *WARS* could play a role in the regulating the embryonic immune response. Besides, EV 0–7 treatment might protect the embryos compared to control, with the latter suffering the damage related to in vitro culture conditions to a greater extent, as well as having a higher *WARS* expression.

Sirtuin 1 (*SIRT1*) was downregulated in a previous study with bovine embryos treated with EV [33]. Besides, extended embryo survival over time was shown in EV-treated embryos [32]. The enhanced embryo development, survival, and the downregulation of *SIRT1* in EVs-treated embryos suggests the possibility of a post-transcriptional downregulation of *SIRT1* in EV-treated embryos by miRNAs [83]. In this way, *SIRT1* in embryos could be potentially targeted by miR-21-5p or miR-9-5p present in bovine oEVs and in porcine oEVs [40,41,42,43,44,45,46,47,48,49,50,51,52,53,54,55,56,57,58,59,60,61,62,63,64,65,66,67,68,69,70,71,72,73,74,75,76,77,78,79,80,81,82,83,84,85]. In the present study, although SIRT1 was not among the DEGs, a higher gene expression was observed in control embryos vs. EV 0–2, as RNA-seq and qPCR results demonstrated. Owczarz et al. [86] showed that overexpression of miR-9 is associated with downregulation of SIRT genes in human peripheral blood mononuclear cells, while the inhibition of miR-9, which was induced the earliest during embryonic stem cell differentiation, prevented SIRT1 downregulation [87]. Additionally, the SIRT1 protein has been identified as being highly expressed in embryonic stem cells (ESC) and post-transcriptionally downregulated during differentiation [87]. These findings might be associated with a higher cell differentiation status in EV-treated embryos, supported by the increased number of trophectoderm cells compared to controls found in bovine EV-treated embryos [57].

HERPUD family member 2 (*HERPUD2*) was found to be downregulated in E2C and potentially targeted by miR-21-5p or miR-7-5p present in poEVs. It has been reported that stressful in vitro conditions such as oxygen and glucose deprivation downregulated miR-7, which upregulated Herpud2 mRNA, and in this way, other stress proteins in astrocytes [88]. HERPUD1, another member of the HERPUD family, mediated cytoprotective effects against oxidative stress [89]. Immunostaining for HERPUD1 in dying embryos revealed the accumulation of these stress-response products [90]. This leads to the hypothesis that the downregulation of *HERPUD2* in EV-treated embryos, potentially due to miRNA regulation (e.g., mir-7-5p in poEVs), might protect the embryos from stressful IVC conditions and enhance their development in vitro. In this line, Fang et al. [56] suggested that oEVs from in vitro origin could minimize the oxidative damage associated to in vitro environment.

Triosephosphate isomerase 1 (*TPI1*), identified as being downregulated in both E2C and E7C, and potentially targeted by nine different miRNAs in poEVs, has been reported to be altered in IVC embryos cultured under induced oxidative stress conditions [91]. A lower expression of glycolytic enzymes, such as TPI1, has been associated with efficient oxidative activity in surviving blastocysts under stress conditions. Furthermore, it has been shown that miRNAs associated with glycolysis, miR-15a-5p and mir-16-5p, could directly target TPI1 [92]. Altogether, TPI1 alteration in EV-treated embryos might be part of the response to overcome in vitro culture stress conditions [93].

PGAM family member 5, mitochondrial serine/threonine protein phosphatase (*PGAM5*), was identified as being downregulated in E7C and potentially linked to miR-7977 or miR-21-5p contained in poEVs. This gene is involved in various stress responses, from mitochondrial quality control to cell death [94]. The overexpression of PGAM5 has been shown to trigger mitophagic cell death [95]. Besides, an increased PGAM5 expression has been related to aging, with higher PGAM5 expression in in vitro human aged cumulus cells, in murine aged oocytes, and in the cumulus cells of older women compared to their young counterparts [96]. This has been associated with a higher mitochondrial fragmentation that leads to apoptosis, causing imbalance of mitochondria dynamics and affecting the energy capacity of mitochondria in cells. Thus, reducing PGAM5 expression has been suggested as a way to ensure proper oocyte and mitochondrial activity, to avoid oxidative damage and energy deficiency, and thus, slow down the aging process [96]. On the other hand, miR-21-5p has been shown to directly suppress mitophagy and mitochondrial damage by targeting PGAM5 [97]. Altogether, the decreased expression in EV-treated embryos could be regulated by miR-21-5p contained in poEVs and might protect the mitochondrial function and metabolism in EVs-treated embryos.

Finally, we would like to mention that the characterization of oEVs has been performed only morphologically by TEM in this study, due to the limited amount of EVs obtained from OF samples that were used for TEM analysis, protein concentration, oEV supplementation during IVC in two different treatments, as well as RNA-seq analysis. We considered it important to examine the oEVs and OF on embryo development from the same oviductal sources, which limited also the OF availability for oEV isolation. The characterization of the porcine oEVs can be found in recent publications from our laboratory using exactly the same protocol as here: (1) morphologically by TEM and EVs size distribution, showing that the EV population in porcine OF mostly consists of exosomes (83%; 40–150 nm) and fewer microvesicles (17%; >150 nm); (2) molecularly by Western Blotting for known exosomal markers (TSG101, FLOT1 and annexins 1, 4, and 5) and other well-known oviductal proteins (OVGP1, MYH9, and HSPA1A) [25]; (3) functionally, showing the effect of oEVs on sperm functional parameters and fertilization.

## 5. Conclusions

The present study demonstrated that supplementation with oEVs enhances porcine in vitro embryo development and modulates the embryonic transcriptome, reflecting the maternal impact on the embryo at the molecular level. Besides, the results emphasize the importance of mimicking the maternal environment during IVC, with the EV 0–2 treatment providing higher cleavage rates as well as blastocyst rates. An integrative analysis of poEV cargo and embryonic RNA-derived alterations point to specific RNAs and miRNAs that could be crucial in supporting embryo development: by regulating cell differentiation and embryo survival, providing cytoprotective effects against oxidative stress, and modulating mitochondria dynamics and energy metabolism. Overall, the data imply that oEVs might be the key factors of OF responsible for supporting embryo development in the oviduct and point to them as a strategy to improve porcine embryo IVP. Moreover, it provides a molecular basis for further studies investigating potential intervention strategies in porcine IVP.

## Figures and Tables

**Table 1:** Primer sequences for genes used in gene expression analysis by quantitative real–time (qPCR).

Genes	Primer Sequence 5′ to 3′	Product Length, bp	Annealing Temp, °C	Accession No.	Ssc Gene ID
*S100A11*	F: ATGCTGGAAGGGACGGTAAC	121	64	NM_001004045.1	445534
	R: ATCATGCGGTCAAGGACACC				
*ANXA2*	F: CGGCAAGTCCCTGTACAACT	171	60	NM_001005726.1	406192
	R: TGCAGTTAGTCGCAAGCTGA				
*GABARAPL1*	F: ACACCATCCCTCCCACTAGC	99	59	NM_001190287.1	100462751
	R: AGACACTCTCGTCGCTGTAG				
*HSPA5*	F: GAAACCGTGGGAGGTGTCAT	146	64	XM_001927795.7	407060
	R: TCTTTTGTCAGGGGTCGCTC				
*WARS*	F: TGGACGTGTCCTTCATGTACC	132	59	XM_003128728.6	100515390
	R: GCTGTAGCACCTCTATGAGCA				
*SIRT1*	F: GAACCGATGGAGAGTCCAGG	196	64	NM_001145750.2	751859
	R: ATACCTCAGCGCCATGGAAA				
*RN18S*	F: ATACATGCCGACGGGCGCTG	84	64	NR_046261.1	100861538
	R: GGGAGGAGGCTGACCGGGTT				
*UBB*	F:GCAGCTGGAAGATGGCCGCA	88	60	NM_001105309.1	100125968
	R: GCAGCCACCCCTCAGACGGA				
*H3-3A*	F: CTTCCAGCGTCTGGTGCGGG	200	59	NM_213930.1	396970
	R: CTCCACGTATGCGGCGTGCT				

**Table 2:** Effect of oviductal secretions during in vitro embryo culture on cleavage rate.

Treatment During IVC	No. Oocytes	Cleavage Rate Day 2 (%)
Control	213	44.9 ± 0.8 ^a^
OF 0–2	220	46.6 ± 1.3 ^ab^
OF 0–7	220	45.8 ± 1.3 ^ab^
EV 0–2	221	51.8 ± 2.1 ^b^
EV 0–7	219	50.5 ± 2.1 ^ab^

OF: Oviductal fluid supplementation; EV: Oviductal extracellular vesicles supplementation; Control (without supplementation); OF 0–2 and EV 0–2 (during the first 2 days of IVC); OF 0–7 and EV 0–7 (during the first 7 days of IVC) (Mean ± SEM). Different letters on the same column indicate significant differences within in vitro treatments (*p* < 0.05).

**Table 3:** Confirmation of RNA sequencing results by qPCR for six selected genes.

Gene	Technology	Embryo Treatment: EV 0–2 vs. CO		Embryo Treatment: EV 0–7 vs. CO		EV Cargo
		log FC EV 0-2/CO *	PValue RNA-seq PValue qPCR	log FC EV 0–7/CO *	PValue RNA-seq PValue qPCR	CPM RNA-seq Cq qPCR
*S100A11*	RNA-seq	0.83	0.0004	0.79	0.0009	845.40
	qPCR	4.97	0.0022	5.89	0.0018	24.24
*ANXA2*	RNA-seq	1.03	0.0007	0.78	0.0140	665.07
	qPCR	1.87	0.0290	1.37	0.3010	23.67
*GABARAPL1*	RNA-seq	1.01	0.0025	1.07	0.0013	128.20
	qPCR	9.75	0.0050	1.04	0.0936	27.36
*HSPA5*	RNA-seq	0.04	0.9207	−1.07	0.0076	120.76
	qPCR	−1.61	0.1725	−1.24	0.0537	27.70
*WARS*	RNA-seq	−0.37	0.1955	−1.47	0.0000	71.56
	qPCR	1.58	0.6052	−13.17	0.0310	28.71
*SIRT1*	RNA-seq	−0.41	0.3391	−0.60	0.2188	28.70
	qPCR	−3.88	0.1772	0.24	0.9055	31.55

* Mean-centered log2 expression values were used to compare data of the relative expression levels of RNA seq and qPCR results. Statistical differences are represented by *p*-value for the RNA-seq data and qPCR data (*p* < 0.05).

## Data Availability

RNA-seq data have been deposited at NCBI’s Sequence Read Archive (SRA) under the BioProject accession ID PRJNA847780 (https://www.ncbi.nlm.nih.gov/sra/PRJNA847780, accessed on 19 July 2022).

## Supplementary Materials

The following supporting information can be downloaded at: https://www.mdpi.com/article/10.3390/biom12091300/s1, Figure S1: Principal component analysis of all genes (A) and miRNAs (B) identified across all embryo samples (EdgeR multi-dimensional scaling plot, https://bioconductor.org/packages/release/bioc/html/edgeR.html, accessed on 19 May 2022) [44]. Sample ID: embryo pool number, treatment, and replicate; Supplementary Data S1: Transcriptomics datasets of embryos and EVs.xlsx and Supplementary Data S2: Integrative data analysis of embryos and EVs.xlsx.
