# Supplementary figures and images for: Oviductal Extracellular Vesicles Enhance Porcine In Vitro Embryo Development by Modulating the Embryonic Transcriptome
Source: Biomolecules. 2022 Sep 15;12(9):1300. doi: 10.3390/biom12091300 (PMC9496104; doi:10.3390/biom12091300)

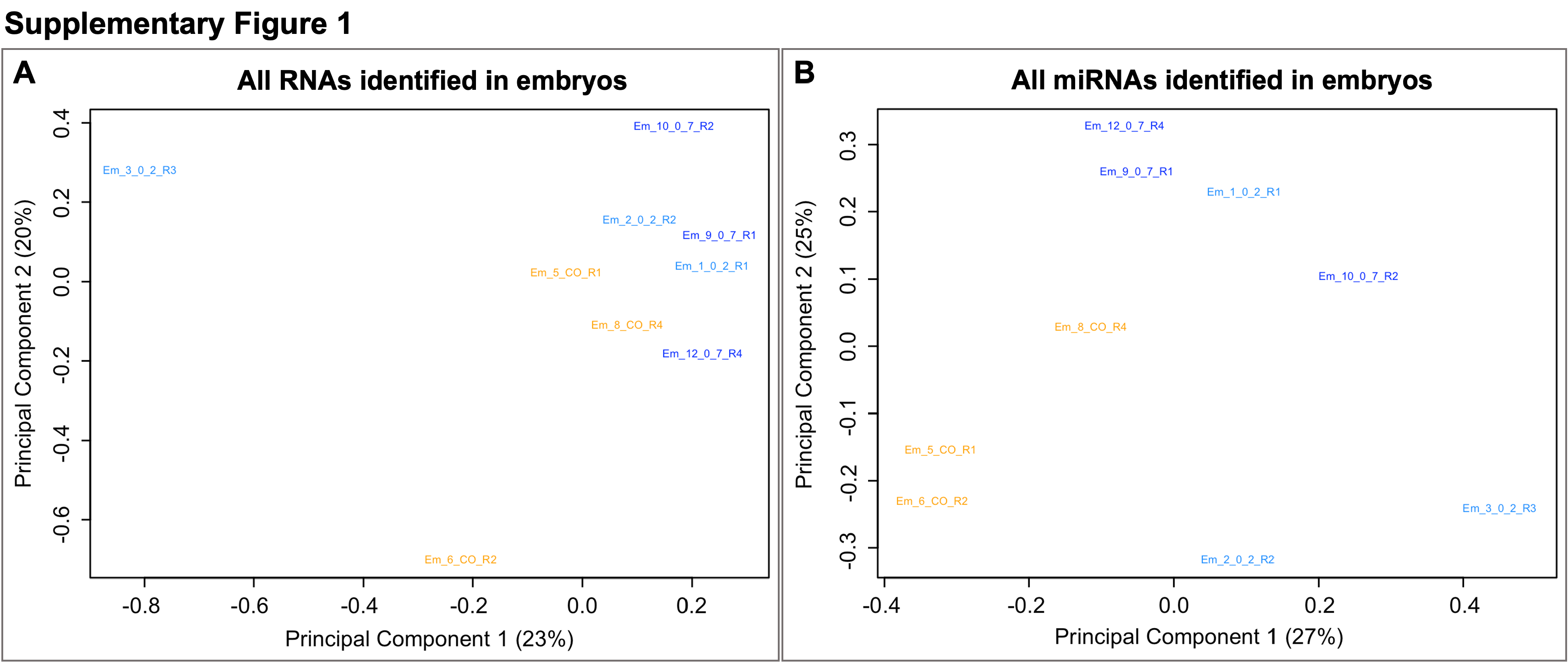

Supplement: Supplementary file 1 [file biomolecules-12-01300-s001.zip › Figure S1.tif]
